# Leader Cells: Invade and Evade—The Frontline of Cancer Progression

**DOI:** 10.3390/ijms251910554

**Published:** 2024-09-30

**Authors:** Brittany R. Doran, Laura R. Moffitt, Amy L. Wilson, Andrew N. Stephens, Maree Bilandzic

**Affiliations:** 1 Hudson Institute of Medical Research, Clayton 3168, Australia; brittany.doran@monash.edu (B.R.D.); laura.moffitt@hudson.org.au (L.R.M.); amy.wilson@hudson.org.au (A.L.W.); andrew.stephens@cleodx.com (A.N.S.); 2 Department of Molecular and Translational Sciences, Monash University, Clayton 3168, Australia

**Keywords:** leader cell, collective invasion, cancer, keratin 14, chemoresistance, metastasis

## Abstract

Metastasis is the leading cause of cancer-related mortality; however, a complete understanding of the molecular programs driving the metastatic cascade is lacking. Metastasis is dependent on collective invasion—a developmental process exploited by many epithelial cancers to establish secondary tumours and promote widespread disease. The key drivers of collective invasion are “Leader Cells”, a functionally distinct subpopulation of cells that direct migration, cellular contractility, and lead trailing or follower cells. While a significant body of research has focused on leader cell biology in the traditional context of collective invasion, the influence of metastasis-promoting leader cells is an emerging area of study. This review provides insights into the expanded role of leader cells, detailing emerging evidence on the hybrid epithelial–mesenchymal transition (EMT) state and the phenotypical plasticity exhibited by leader cells. Additionally, we explore the role of leader cells in chemotherapeutic resistance and immune evasion, highlighting their potential as effective and diverse targets for novel cancer therapies.

## 1. Introduction

Metastasis is a complex, multi-step process that facilitates the dissemination of cancer cells from a primary tumour to peripheral sites and is the leading cause of cancer-related deaths across all malignancies [1]. Despite the identification of key genetic mutations and signalling pathways involved in promoting metastasis, the precise mechanisms by which these drivers facilitate spread are not fully elucidated. Numerous studies have identified essential mediators of epithelial–mesenchymal transition (EMT), enabling cancer cells to acquire migratory and invasive properties [2]; however, the mechanisms underlying the transition of epithelial cells during malignant transformation, in a cancer type- and stage-specific manner, remain an active area of investigation [3].

Large scale coordinated cellular translocation is a fundamental process enabling the establishment and complex organisation of tissues and organs during embryogenesis and is essential for wound healing and angiogenesis in adult tissues [4,5]. Epithelial cells exist as large, immobile sheets maintained by stable, E-cadherin-based adherens junctions with neighbouring cells [6]. Under normal circumstances, signals promoting relocation induce epithelial cells to undergo single-cell migration by acquiring a “mesenchymal” programme. This process is driven by a powerful set of transcription factors including the Zinc finger E-box binding homeobox (*ZEB*) family, Snail (*SNAI1*), Slug (*SNAI2*), and Twist Family BHLH Transcription Factor 1 (*TWIST1*) [7]. The transcriptomic shift abolishes epithelial features, including stable cell–cell and cell–matrix adhesions, induces front–rear polarity, and confers cells with an increased migratory and invasive propensity [7]. This phenotypic change is referred to as EMT. EMT facilitates the translocation of single cells; however, in the context of organ assembly and embryogenesis, which require bulk transport of cells, EMT is relatively inefficient [8].

A specialised process called collective migration (also referred to as collective invasion) allows the “global movement” of epithelial cells without the requirement of EMT [9]. Instead, epithelial morphology is largely preserved, and cell–cell adhesions are maintained, allowing the migrating cluster to collectively traverse the surrounding extracellular environment as a cohesive group [4]. While a complete EMT is not a prerequisite for collective migration, the cohort’s constituents become phenotypically and functionally heterogenous and are classed as one of two cell types primarily based on their location within the cluster [10,11]. A “Leader cell” (LC) refers to a cell localised to the front of the cluster’s leading edge, actively directing migration [10,12]. The remaining cells representing the majority of the cluster are “Follower cells” (FC) and trail LCs interacting with the ECM and exerting traction force to a lesser extent than LCs [10,12,13]. Conversely, LC traits such as pathfinding, traction force, and matrix remodelling are not exclusive to cells at the front; consequently, LCs have the potential to drive directional migration from any position within the cluster. As collective migration offers an efficient means of transporting large groups of epithelial cells over long distances [4], it is unsurprising that this developmental process is co-opted by cancer cells during metastasis. LCs driving cancer dissemination have been identified across multiple malignancies [14,15,16,17,18,19]. To date, research has focused on the fundamental role of LCs in collective invasion, with recent findings indicating that LCs may contribute more significantly to cancer progression. In particular, LCs are recognised as a treatment-resistant cancer cell subset with the potential to drive disease recurrence [20,21].

## 2. Leader Cell-Driven Mechanisms in Collective Cancer Cell Invasion

**Figure 1:**
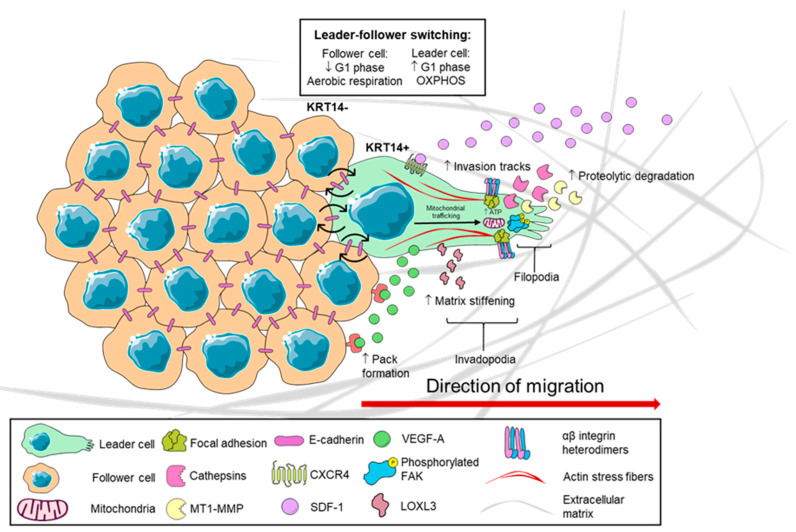
Leader cells drive collective cancer invasion. Leader cells (LC) at the front of multicellular cancer clusters drive collective invasion. LCs, characterised by front-rear polarity and protrusions such as invadopodia and filopodia, use αβ integrins to attach to the ECM and propel the cluster forward. Due to high energy demands, LCs exchange roles with adjacent FCs which in turn adopt the LC phenotype. Mitochondrial trafficking to invadopodia supports energy requirements. LCs enhance invasion by secreting LOXL3 for ECM stiffening, fibronectin for FAK phosphorylation, and proteases for ECM degradation. LCs also release VEGF-A to maintain cluster integrity and respond to chemokine gradients like SDF-1 through CXCR4. Figure components adapted from Servier Medical Art. MT1-MMP: Membrane type 1-matrix metalloproteinase, CXCR4: CXC-motif chemokine receptor 4, SDF-1: Stromal-derived factor-1, VEGF-A: Vascular endothelial growth factor A, FAK: Focal adhesion kinase, LOXL3: Lysyl oxidase-like 3, ECM: Extracellular matrix, KRT14: Keratin 14. Up arrow represents increased and down arrow represents decreased.

Phenotypically, LCs exhibit an elongated, spindle-shaped polarized morphology and undergo dynamic cytoskeletal rearrangements, including the formation of large, actin-based membrane projections extending from the leading edge into the immediate extracellular environment [4,22,23]. By contrast, FCs lack large, distinct membrane protrusions and instead retain a traditional epithelial morphology [4,18,24]. This morphological organisation confers both the individual LC, and the entire migrating cluster, with a front–rear polarity axis that enables locomotion [25]. Cancer cells within a collectively migrating cluster are coupled to their neighbours via cadherin-based adherens junctions acting as conduits for the transmission of LC-generated force via actomyosin cables contributing to movement as a single cellular unit [26,27] (Figure 1). This mechanical coupling via E-cadherin inhibits FCs from generating LC-like protrusions [28].

As LCs are located at the front of the cluster, they possess a membrane “free-edge” that houses heterodimeric, transmembrane integrin α and β subunit pairs, particularly β1 heterodimers [4], used to directly engage with extracellular matrix (ECM) components including fibronectin and laminin [29]. Upon integrin/ECM engagement, complex signalling cascades lead to the establishment of focal adhesions at the leading edge, activating members of the Rho family of small GTPases [23,26,30,31]. The Rho GTPases, cell division cycle 42 (Cdc42), and Rac at the LC leading edge induce the rapid polymerisation of F-actin filaments that push the cell membrane outwards [26], forming large membrane protrusions called invadopodia and smaller protrusions called filopodia that interact with the ECM [32]. Upon activation, the Rho GTPase RhoA acts on Rho Associated Coiled-Coil Containing Protein Kinase (ROCK), a kinase that phosphorylates myosin regulatory light chain 2 (MLC2), ultimately leading to the contraction of actomyosin cables that span the entirety of the cell cluster [29]. Actomyosin contraction is the major force-generating step that allows LCs to physically pull the trailing FCs forward along the migration axis [27].

As the key orchestrators of the collective invasion, LCs facilitate cohesive movement through additional roles. Their position at the front of the cluster enables them to respond to chemokine gradients such as stromal-derived factor 1 (SDF-1) via receptor CXCR4 [33,34], honing the movement of the invading cluster towards the stroma. Additionally, LCs secrete Vascular Endothelial Growth Factor-A (VEGF-A) which acts on FCs, stimulating them to maintain “pack formation” ensuring that the migrating cluster remains cohesive [35]. Although LCs are the primary drivers of collective invasion, cooperation with FCs is essential for migration and the efficient invasion of target tissue [13]. FCs contribute to this process through restriction of leadership to the leading edge, promoting migration in a directional manner [27,36] and facilitating LC growth by rescuing them from mitotic defects such as cytokinetic instability via secreted factors that remain unidentified [35]. FCs also actively drive the direction of the invading cluster by extending cryptic lamellipodia under LCs [37], and suppression of the VEGF receptor in LCs results in FCs assuming control [38].

The ECM is composed of collagen fibres, laminin, proteoglycans, and fibronectin [39], forming a physical structure preventing cancer cells from metastasising. However, its composition is highly tissue- and organ-specific, influencing how LCs interact with the ECM [40]. During tumour progression, ECM components in regions adjacent to the tumour undergo dynamic changes, including alterations in mechanical properties such as rigidity and density, as well as mediating changes in the repertoire of ligands recognized by receptors on LCs, like integrins [41]. These changes impact LC behaviour, facilitating invasion and metastasis through diverse ECM–LC interactions [42]. LCs manipulate the local ECM to promote migration by secreting fibronectin [35] and engage with these fibres by LC-expressed integrins inducing the formation of enlarged adhesion sites characterised by high levels of phosphorylated Focal Adhesion Kinase (FAK) Y397 [35]. LCs also exert mechanical forces that remodel the ECM and clear “migration tracks” permitting easier passage for trailing FCs [14,43]. As the actomyosin cables in LCs are indirectly attached to the ECM via integrins, their contraction results in the exertion of force that contributes to the remodelling of the ECM [29]. In densely packed ECMs, additional enzymatic degradation is achieved through the delivery of proteases including cathepsins or matrix metalloproteinases (MMPs) such as Membrane Type 1-MMP (MT1-MMP) to LC invadopodia. Stromal cells, such as cancer-associated fibroblasts, also contribute to ECM remodelling using similar mechanisms that drive the expression of LC-associated genes to further promote collective invasion [44,45].

## 3. Extracellular Matrix Density Regulates Leader Cell Emergence

The ECM of the tumour microenvironment (TME) is characterised by the presence of thick, cross-linked collagen fibres with increased ECM stiffness [46]. Breast cancer LCs displaying a highly invasive, protrusion-rich morphology are strongly associated with regions of ECM containing high abundance collagen I fibrils compared to those with a less invasive phenotype [15]. Compressive stress exerted by the ECM onto tumour cells as a result of uncontrolled proliferation enhances the localised secretion of fibronectin and in turn increases the number and invasiveness of LCs [47]. Reciprocally, LCs directly contribute to matrix stiffening through the deposition of the collagen crosslinking protein Lysyl Oxidase Like 3 (LOXL3) [48].

Vinculin is a force-responsive protein that stabilises focal adhesions and is highly expressed at the invasive front of polyoma middle T oncoprotein (PyMT) mammary tumours where the ECM is the stiffest [49]. 3D culture of mammary epithelial cells under stiff ECM conditions increases the amount and activation of vinculin at focal adhesions, along with enhancing the recruitment of Akt^p473^ and FAK^p397^ and the formation of stable vinculin–talin–actin scaffolding complexes supporting phosphoinositide 3-kinase (PI3K) signalling and tumour invasion [49]. Interestingly, disrupting the deposition of lysyl oxidases can reverse ECM stiffening [50]. Furthermore, prevention of collagen crosslinking in the premalignant epithelium has also been shown to reduce integrin clustering, decrease focal adhesion formation, and dampen PI3K signalling leading to increased tumour latency and smaller, low-grade lesions in vivo [50]. Therefore, ECM rigidity directly regulates the invasive and highly dynamic nature of LCs, and disrupting the stiffening of tumour-associated ECM may offer an avenue to modulate LC emergence.

## 4. The Leader–Follower Cell State Is Dynamic and Engages in Metabolic Reprogramming

Invasion of cancer cells through dense ECM has substantial energetic requirements. Cancer cells typically rely on aerobic glycolysis as their primary energy source [51]. However, in order to penetrate through dense ECM, LCs are faced with high energetic demands overcome by an increased uptake of glucose and reliance on mitochondrial respiration, as a more efficient source of energy generation [51,52]. Resultingly, LCs have a clear metabolic preference for mitochondrial oxidative phosphorylation (OXPHOS) while FCs rely on glycolysis as their primary energy source [53,54]. The energy expenditure required for invasion is directly related to ECM density (i.e., higher energetic demands in denser matrices) [55]. As invasion is an energy expensive process, once the energy level of a LC is depleted below a threshold, exhausted LCs are swapped out for nearby FCs that acquire the LC phenotype [56]. The dynamic, “relay-like” leader–follower swapping sustains the forward momentum required for invasion through complex and dense ECMs [56].

Leader–follower cell switching is also regulated by cell cycle stage during breast cancer invasion. Bayamagnai and colleagues demonstrated that LCs predominantly occupy the G1 phase when engaging in active invadopodia-driven ECM degradation [57]. Specifically, G1 phase LCs upregulated the expression of key invadopodia components MT1-MMP and cortactin and translocated scaffold protein Tks5 to invadopodia [57]. As LCs transition from G1 to G2, they retreat from the leading edge and are replaced by another G1-occupying LC [57] suggesting that cell cycle stage dictates leader–follower cell status. These studies demonstrate that the LC state is a transient, rapidly cycling phenotype that responds to the bioenergetic demands of the TME.

Targeting LCs via exploitation of their metabolic dependencies has previously been investigated. The inhibition of OXPHOS in LCs in vitro coincides with an increase in the production of lactate and impaired 3D collective invasion [53]. In vivo, treatment with a mitochondrial complex V inhibitor, oligomycin, had no impact on LC viability, but significantly reduced the number of lung metastases in orthotopic human breast cancer models [54], indicating that OXPHOS is a requirement of LC-mediated collective invasion. Furthermore, other inhibitors of mitochondrial respiration including rotenone, antimycin A, and IACS-10759 have also been used to explore the relationship between OXPHOS and collective migration [58,59].

As energy generation is necessary for cell survival, this indicates that LCs surviving OXPHOS inhibition must undergo metabolic reprogramming to utilise glycolysis instead [54]. Conversely, treatment of FCs with a glycolysis inhibitor was found to reduce proliferation and increase invasiveness [53], characteristics normally displayed by LCs providing evidence of bi-directional metabolic reprogramming. Dual inhibition of glycolysis and OXPHOS acts synergistically and inhibits both cell proliferation and invasiveness [53].

Metabolic reprogramming is also evident on the subcellular level in LCs. The formation and maintenance of the protrusive structures at the leading edge requires a high degree of energy expenditure; therefore, to provide a potent and localised source of ATP to sustain invasion, LCs traffic mitochondria directly to the leading edge [53,60]. This process is regulated by AMP-activated protein kinase (AMPK) sensors in SKOV3 ovarian cancer cells that initiates microtubule-mediated trafficking of mitochondria to LC invadopodia to produce sufficient ATP levels to sustain invasion [60]. Targeting the Inhibitor of Apoptosis (IAP) family protein survivin in PC3 prostate cancer cells impaired mitochondrial respiration and ATP production, disrupted trafficking of mitochondria to membrane protrusions and reduced invasiveness in vitro [61]. While disruption of subcellular mitochondrial recruitment to the leading edge has been demonstrated to impair invasive abilities in vitro [60,62,63,64], the effect of this inhibition on metastatic deposition in vivo has not been investigated.

## 5. Leader Cells Are Defined by Expression of Basal Epithelial Marker Keratin 14 (KRT14)

Keratin 14 (KRT14) is a conserved epithelial marker of the LC subset across many epithelial malignancies including breast, bladder, ovarian, and lung cancers [15,17,65,66,67,68]. KRT14 is a type I cytokeratin that is co-expressed as a heterodimer with the type II cytokeratin Keratin 5 (KRT5) to form intermediate filaments [69]. Early studies established the role of KRT14+ LCs in collective invasion across several breast cancer subtypes [15,65]. Although MMTV-PyMT tumour organoids initially display low numbers of KRT14+ cells, the formation of invasive tumour strands is proceeded by the acquisition of KRT14 expression at the leading edge [15]. KRT14+ LCs also localise to the outer edges of ovarian cancer spheroids in a similar manner and mediate attachment to and invasion of the mesothelium [17] with KRT14 expression restricted to cancer cells at the invasive interface [17]. Furthermore, the proportion of KRT14+ LCs is significantly enriched in breast cancer micro-metastatic tumours compared to relatively low numbers observed in the primary tumour and established secondary metastases (43–67% vs. 0.9% and 2.5%, respectively) [65]. Polyclonal, LC-led collective invasion was identified as the primary mode of metastasis in vivo by increasing metastatic efficiency >100 fold compared to single-cell dissemination [65]. Moreover, bulk RNA-sequencing showed that KRT14 transcript levels were correlated with the expression of metastatic niche remodelling and metastatic cell survival genes (*Tnc*, *AdamTs1*, *Jag1*, *Birc5*) [65]. Lung cancer KRT14+ LCs co-express gastrokine 1 (Gkn1) which enhances their metastatic potential by supressing Akt and inhibiting cell death via anoikis [68]. Furthermore, the KRT14-expressing subset of papillary non-muscle-invasive upper tract urothelial carcinomas displayed an enrichment of genes associated with “cellular movement”, “adhesion”, and “cellular growth and proliferation” and the downregulation of genes associated with “cell death” [70].

Numerous studies have demonstrated that disruption of KRT14 expression inhibits collective invasion in vitro and in vivo [17,20,71]. Ex vivo breast cancer and salivary adenoid cystic carcinoma 3D culture assays demonstrated that KRT14 knockdown abrogated the appearance of multicellular invasive strands [15,20]. Similarly, CRISPR-mediated KRT14 knockout in ovarian cancer cell lines impaired cancer cell migration and invasion with no effect observed on cell viability or proliferation [17]. Conversely, KRT14 overexpression increased migration of SACC-83 salivary adenoid cystic carcinoma cells compared to wild-type controls [20] and enhanced the formation of OVCAR4 3D spheroids followed by rapid outgrowth with no effect on proliferation [17]. In MMTV-PyMT breast cancer organoids, KRT14 knockout was shown to have no impact on invasion into collagen I substrate, with invasive behaviour linked to the mechanoresponsive activation of yes-associated protein-1 (YAP) [72]. Although KRT14 expression is closely associated with the LC phenotype, its functional contribution to collective invasion is not completely understood.

KRT14 expression is also a requirement for metastatic dissemination in vivo. Several breast cancer mouse models have shown that shRNA-mediated KRT14 knockdown or diphtheria toxin-mediated ablation of KRT14+ tumour cells resulted in smaller primary tumours and significantly fewer metastatic lesions [65,71].

## 6. Potential Mechanisms of KRT14 Involvement of Leader Cell-Driven Collective Invasion

As a prototypical epithelial marker, the functional role of KRT14 in LC-mediated collective invasion is not understood. Intermediate filaments (IFs), including keratins, provide structural protection from deformations imposed on cells when migrating through complex ECMs and can interact with components of the actin cytoskeletal network [73]. Vimentin is an IF expressed in mesenchymal cells whose influences on cellular migration has been well characterised [74,75,76]. Vimentin controls the organisation of the actomyosin network, promoting actin-based membrane protrusions at the leading edge through phosphorylation-mediated interactions with small Rho GTPases, influencing the formation of focal adhesions and regulating the expression of EMT-related factors [77,78,79,80]. Interactions between KRT14 and vimentin have been reported as having important roles in regulating migration in epithelial cells [81,82]. Vimentin co-localises with KRT14 at the leading edge of epidermal keratinocytes and a physical interaction between KRT14 and vimentin had been evidenced via co-immunoprecipitation assays [82]. Mutation of the vimentin-KRT14 binding site disrupted this interaction and resulted in the impaired migration of keratinocytes [82]. In addition, the suppression of vimentin in oral squamous cell carcinoma cells corresponds with the depletion of KRT14 and KRT5 and reduced tumorigenicity, potentially through vimentin-mediated ΔNp63 silencing, as an upstream regulator of KRT14 [81].

Other cytokeratins have been reported to drive cell migration. The phosphorylation of KRT8 at Ser431 by sphingosylphosphorylcholine (SPC) induced keratin reorganisation and increased the number of focal adhesions at the leading edge, enhancing tumour cell migration in pancreatic cancer cells [83]. Studies examining the depletion of IFs including KRT8/KRT18 or nestin reported alterations in integrin signalling along with plasma membrane localisation and distribution of phosphorylated FAK at focal adhesion sites corresponding to changes in cancer cell migratory and invasive abilities [84,85,86].

Epidermolysis bullosa simplex (EBS) is a disease arising from inherited missense mutations in *KRT14* or *KRT5* genes [87] and is characterised by severe skin blistering in patients. Basal epidermal keratinocytes harbouring the most common underlying EBS mutation (KRT14_R125_) displayed weaker traction forces and a reduction in RhoA activity resulting in immature focal adhesions that did not localise to the leading edge [87]. The resultant cells migrated at a lower velocity and lacked persistent directional movement, suggesting that KRT14 may control collective invasion through regulation of RhoA activity [87].

## 7. Leader Cells Occupy Diverse Hybrid-EMT States across Cancer Types

A number of studies have classified LCs as occupying a hybrid-EMT state as they express some, but not all, of the hallmark EMT transcription factors (e.g., TWIST1, Snail, vimentin), in combination with typical epithelial markers such as KRT14 and p63 [15,18,24,66]. However, the composition of EMT transcriptional programmes expressed by LCs varies widely between cancer types and subtypes [15,24,35,66,88,89]. LCs across three lung cancer cell lines (A549, H1975, and H1299) displayed upregulation of EMT genes including *SNAI2*, *TWIST1*, *ZEB1*, *ZEB2*, *TNNB1*, *NFKB1*, *NFKB2*, and *AKT1*, yet *SNAI1* and *VIM* were the only two EMT genes consistently upregulated across all LCs [18]. Invasive luminal B breast cancer LCs were reported to express epithelial markers KRT14 and p63 but lacked expression of typical EMT markers TWIST1, Slug, and vimentin [15]. Conversely, MCF7 luminal A breast cancer cells leading collective invasion co-expressed E-cadherin, N-cadherin, ZEB1, Snail, Twist, and vimentin [24]. Ovarian cancer leader cells displayed enriched expression of *KRT14* and *TWIST-1* but expression levels of N-cadherin, vimentin, Slug, and Snail remained identical to FCs [66]. Therefore, the hybrid-EMT phenotype displayed by LCs is highly variable and heterogenous between malignancies and subtypes; however, it appears stable rather than transitory during the metastatic process. Furthermore, although LCs are central to EMT, particularly in cancers such as breast and ovarian cancer, emerging evidence also suggests that LCs influence the reverse process, mesothelial-to-epithelial transition (MET) [90]. MET is critical for the colonization and establishment of metastatic sites [7,91]. The plasticity of LCs is a key factor in these processes, allowing for dynamic shifts between epithelial and mesenchymal phenotypes depending on the stage of metastasis [92]. This plasticity enables LCs to adapt to changing microenvironmental cues, facilitating both the initial invasion and the subsequent re-epithelialization required for metastatic outgrowth [91]. Furthermore, using breast cancer models, Cheung et al. elegantly showed that once distant metastatic deposits are established, LCs undergo MET and/or phenotypic reversion to form proliferative metastatic colonies.

## 8. Leader Cells Display Phenotypic Plasticity and Stem-like Features

Cancer cells in a hybrid-EMT state often exhibit increased stemness phenotypes, drug resistance, and immunoregulatory properties [7,11,24]. LCs have emerged as a stem-like population comprising a gene expression profile encompassing several stem markers including ALDH1A1, CD44v6, SOX2, Nanog, and TWIST-1 [66,88,93,94]. LCs possess high sphere-forming potential and are inherently resistant to chemotherapy [66,67]; accordingly, LCs have been compared with cancer stem cells (CSCs) [95,96]; however, several distinctions can be made between the two. Occupying a state of quiescence is a key mechanism used by CSCs to evade chemotherapeutics that target rapidly dividing cells [97,98,99]. LCs do not represent a quiescent population and proliferate at the same [66] or a reduced rate [35] compared to FCs. Furthermore, CD44+CD24− CSCs have been found to be a diverse population compared to LCs, and the two subsets co-exist together within breast cancer [88]. While some studies suggest that LCs across several epithelial cancers possess stem-like features (e.g., self-renewal and resistance to chemotherapy), this may not be a universal trait of all LCs.

The “LC state” also represents a transient phenotype that can be acquired by cancer cells upon exposure to external stimuli, rather than a fixed cellular linage. Acquisition of the LC status occurs independently of cell division as LCs have been shown to arise in the presence of cell division inhibitors [66]. Furthermore, KRT14 acquisition in the absence of cell division has also been demonstrated in breast cancer [15,71]. Single-cell sorting of individual leader (KRT14+) and follower (KRT14−) ovarian cancer cells demonstrated that each gave rise to a mixed leader–follower population that maintained the same proportions of LCs as the initial line from which the cells were derived [66]. As a further example, breast cancer cells sorted into “pure-LC” and “pure-FC” subsets injected into mice formed tumours that reverted to a mixed population that was composed of equal proportions of LCs and FCs of which the cells were derived [88]. LCs can also be induced by the microenvironment as a co-culture of breast cancer cells with M2-polarised tumour-associated macrophages inducing a significant increase in CD44 that corresponded with an increased proportion of the LC phenotype [88]. Additionally, chemotherapy has been shown to enrich the LC subset in bladder and ovarian cancers [66,67], suggesting that the LC phenotype may offer survival advantages for cancer cells.

## 9. Leader Cell-Mediated Immune Evasion

**Figure 2:**
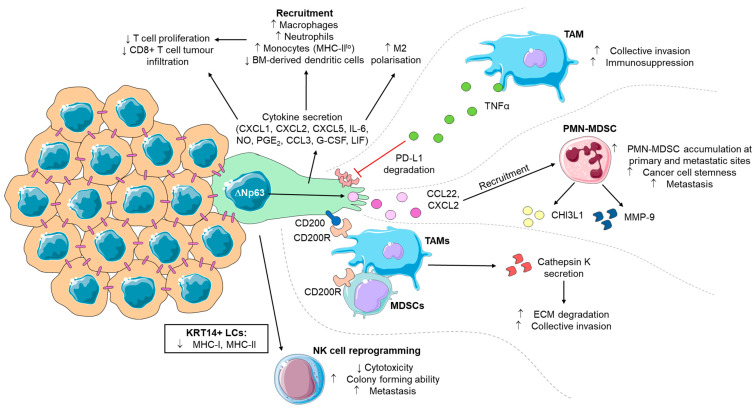
Influences of leader cells on immune subsets. Leader cells interact with immune subsets to promote tumour survival and metastasis. LCs secrete cytokines that can recruit immunosuppressive immune subsets such as TAMs, PMN-MDSCs, neutrophils, and monocytes into tumours coinciding with M2-polarisation of TAMs and impaired T cell proliferation and infiltration. LC-driven collective invasion is enhanced via cell surface CD200-CD200R engagement on TAMs or MDSCs which promotes secretion of cathepsin K to enhance ECM degradation, or, via TNFα-dependent inhibition of PD-L1 degradation in LCs which increases metastatic abilities. LC-directed accumulation of PMN-MDSCs in tumours increases cancer cell stemness and enhances metastasis. Cytotoxic immune subsets, such as NK cells, are also reprogrammed by LCs into a metastasis-supportive phenotype with reduced cytotoxicity. Figure components adapted from Servier Medical Art. BM: Bone marrow, IL-6: Interleukin-6, NO: Nitric oxide, PGE_2_: Prostaglandin E2, G-CSF: Granulocyte colony-stimulating factor, LIF: Leukaemia inhibitory factor, TAM: Tumour-associated macrophage, TNFα: Tumour necrosis factor-alpha, PD-L1: Programmed death-ligand 1, PMN-MDSC: Polymorphonuclear myeloid-derived suppressor cells, CHI3L1: Chitinase-3-like protein 1, MMP-9: Matrix metalloproteinase-9, MDSC: Myeloid-derived suppressor cell, NK: Natural killer. Up arrow represents increased and down arrow represents decreased.

Although LCs are primarily recognized for their role in guiding collective migration and invasion, emerging evidence suggests that they may also play a significant role in tumour cell evasion and subversion of the immune response (Figure 2). Transcriptomic profiling of KRT14+ LCs in breast cancer demonstrates that major histocompatibility complex (MHC) class-II genes are downregulated in LCs. In addition, genes associated with “chemotaxis”, “positive regulation of T cell activation”, and “antigen processing and presentation of exogenous peptide antigen” pathways were also depleted. KRT14 expression in breast cancer LCs is also negatively correlated with cell surface MHC class-I expression [100], indicating that LCs directly engage in immune evasion strategies to increase their chances of successful colonisation.

Relatedly, breast cancer LCs act to dampen the cytotoxicity of natural killer (NK) cells [100]. Prolonged exposure to LCs resulted in reprogramming of NK cells into a metastasis-supportive phenotype with lower cytotoxic abilities and that enhanced colony formation when co-cultured with tumour cells in vitro; and resulted in increased lung metastases in vivo [100].

LCs also play a pivotal role in recruiting immunosuppressive cells to the TME. In bladder cancer, LCs secrete chemokines that attract neutrophils, monocytes with impaired antigen presentation abilities, and tumour-associated macrophages (TAMs) polarised into an immunosuppressive M2 phenotype [101]. As a result, this coincides with impaired T cell activation, reduced T cell proliferation, and poor infiltration into tumours [101]. TAMs may also reciprocally induce the LC phenotype as luminal breast cancer co-cultures with TAMs increased the number of LCs [88].

Immune cells can also interact with LCs to promote collective cancer invasion. LCs have been shown to express immune checkpoint molecules such as PD-L1 and CD200, which interact with TAMs and MDSCs to drive collective cancer invasion [102]. In triple-negative breast cancer, LCs recruit polymorphonuclear myeloid-derived suppressor cells (PMN-MDSCs) through the secretion of CCL22 and CXCL2, which, in turn, mediate the secretion of MMP9 and chitinase-3-like protein-1 (CHI3L1). This upregulation of MMPs not only facilitates invasion but also contributes to the reorganization of the tumour stroma, impeding immune cell infiltration [103]. Collectively, the tumour-supportive microenvironment established by LCs limits the activity of cytotoxic immune subsets, enabling the tumour to evade immune surveillance [100,101,102,103,104,105].

## 10. Leader Cells Are Chemoresistant and Contribute to Disease Recurrence

Our laboratory has demonstrated that ovarian cancer LCs are resistant to many conventional chemotherapeutics including the first-line treatments carboplatin, cisplatin, and paclitaxel, and the poly (ADP-ribose) polymerase (PARP) inhibitors, olaparib and rucaparib [66]. Further, each of these drugs enriched the overall proportion of LCs within the tumour population in vitro [66]. Real time cell analysis demonstrated that the residual population of resistant LCs drove tumour recovery following cisplatin treatment, a response that was absent in a LC-deficient cell line [66].

A similar response was demonstrated in bladder cancer, in vivo, where KRT14+ LC bladder cancer cells were resistant to combined gemcitabine and cisplatin treatment [67]. Furthermore, apoptosis of chemosensitive tumour cells induced the release of prostaglandin E_2_ (PGE_2_) acting on neighbouring resistant KRT14+ LCs, stimulating proliferation and driving tumour repopulation [67]. Importantly, the proportion of KRT14+ LCs progressively increased following successive cycles of chemotherapy leading to diminished responsiveness to treatments [67]. Blocking the release of PGE_2_ via co-administration of celecoxib, a cyclooxygenase-2 (COX-2) inhibitor, restored chemotherapy effectiveness [67].

Clinical data also suggest a conserved LC response to chemotherapy. Ovarian cancer patients with high KRT14 expression have reduced progression-free survival following standard-of-care platinum and taxane-based chemotherapy (HR 1.27; 95% CI 1.07–1.51 *p* < 0.006) [17]. Likewise, immunohistochemical staining of oral cancers demonstrated a significant correlation between high vimentin and KRT14 expression and increased rates of disease recurrence in patients (*p* = 0.001) [81]. Enrichment of KRT14 expression has also been demonstrated in matched tumour tissues from ovarian cancer patients pre- and post-neo-adjuvant chemotherapy [66].

## 11. Conclusions

LCs are a conserved subset of cancer cells present across multiple epithelial malignancies and are associated with a poor prognosis. LCs display an aggressive phenotype characterised as invasive and drug resistant and are representative of a hybrid-EMT state exhibiting a high degree of plasticity. As LCs have been implicated in driving processes mediating disease recurrence, the current evidence proposes that therapeutic targeting of this subset could offer a potential avenue for the development of novel treatment interventions. Furthermore, although expression of KRT14 has been widely linked with the LC phenotype, further research efforts are also required to elucidate its exact functional role in LC biology.
